# Crystal Structure of Patatin-17 in Complex with Aged and Non-Aged Organophosphorus Compounds

**DOI:** 10.1371/journal.pone.0108245

**Published:** 2014-09-23

**Authors:** Sanjeeva J. Wijeyesakere, Rudy J. Richardson, Jeanne A. Stuckey

**Affiliations:** 1 Toxicology Program, Department of Environmental Health Sciences, University of Michigan, Ann Arbor, Michigan, United States of America; 2 Toxicology Program, Department of Environmental Health Sciences, and Department of Neurology, University of Michigan, Ann Arbor, Michigan, United States of America; 3 Life Sciences Institute and Department of Biological Chemistry, University of Michigan, Ann Arbor, Michigan, United States of America; Weizmann Institute of Science, Israel

## Abstract

Patatin is a non-specific plant lipase and the eponymous member of a broad class of serine hydrolases termed the patatin-like phospholipase domain containing proteins (PNPLAs). Certain PNPLA family members can be inhibited by organophosphorus (OP) compounds. Currently, no structural data are available on the modes of interaction between the PNPLAs and OP compounds or their native substrates. To this end, we present the crystal structure of patatin-17 (pat17) in its native state as well as following inhibition with methyl arachidonyl fluorophosphonate (MAFP) and inhibition/aging with diisopropylphosphorofluoridate (DFP). The native pat17 structure revealed the existence of two portals (portal1 and portal2) that lead to its active-site chamber. The DFP-inhibited enzyme underwent the aging process with the negatively charged phosphoryl oxygen, resulting from the loss of an isopropyl group, being within hydrogen-binding distance to the oxyanion hole. The MAFP-inhibited pat17 structure showed that MAFP did not age following its interaction with the nucleophilic serine residue (Ser77) of pat17 since its O-methyl group was intact. The MAFP moiety is oriented with its phosphoryl oxygen in close proximity to the oxyanion hole of pat17 and its O-methyl group located farther away from the oxyanion hole of pat17 relative to the DFP-bound state. The orientation of the alkoxy oxygens within the two OP compounds suggests a role for the oxyanion hole in stabilizing the emerging negative charge on the oxygen during the aging reaction. The arachidonic acid side chain of MAFP could be contained within portals 1 or 2. Comparisons of pat17 in the native, inhibited, and aged states showed no significant global conformational changes with respect to their Cα backbones, consistent with observations from other α/β hydrolases such as group VIIA phospholipase A2.

## Introduction

Patatin is a soluble 40 kDa non-specific plant lipase that is expressed extensively in the tubers of potatoes [1], [2] and other members of the nightshade family [3]. In the potato, patatin has been reported to constitute approximately 40% (by weight) of the protein content of the tuber [4] and possess insecticidal capabilities [5]. Previously, the crystal structure of a recombinant patatin isoform, patatin-17 (pat17), derived from the tubers of heartleaf nightshade (*Solanum cardiophyllum*), has shown this protein to have a modified α/β hydrolase fold containing a central 6-stranded β-sheet sandwiched by 9 α-helices along with a catalytic center composed of a Ser77-Asp215 dyad [6]. This fold and active site configuration is reminiscent of the human cytosolic phospholipase A2 family of enzymes [7], but it is distinct from the traditional Ser-His-Asp/Glu catalytic triads found in serine proteases and esterases such as chymotrypsin and acetylcholinesterase (AChE), respectively [8], [9].

Recently, interest in patatin and its homologues has been heightened by the discovery that a diverse group of mammalian lipases, termed the patatin-like phospholipase domain containing proteins (PNPLAs), encompass catalytic domains with sequence similarity to patatin [10]. Members of the PNPLA family have been implicated as playing a role in the pathogenesis of human lipid storage disorders and fatty liver disease [10]–[12]. One member of the PNPLA family, PNPLA6, which is better known as neuropathy target esterase (NTE), has been of interest due to its role as the putative initiation site in the pathogenesis of a delayed axonopathy induced by certain classes of organophosphorus (OP) compounds [13]. These represent esters derived from phosphoric acid that have been extensively characterized as inhibitors of serine hydrolases such as NTE (PNPLA6) and acetylcholinesterase (reviewed in [13]–[15]).

Pat17 shares 30% sequence similarity and 18% sequence identity to the catalytic domain of NTE, termed the NTE patatin-homology domain (PNTE) [16]. Previously, patatin has been shown to be inhibited by OP compounds such as methyl-p-nitrophenyl-octylphosphonate, as well as the NTE inhibitor diisopropylphosphorofluoridate (DFP) with undetermined potency [5], [17]. Upon inhibition of a serine hydrolase, certain classes of OP compounds (such as the phosphates and phosphonates) can undergo a secondary reaction termed ‘aging’, resulting in the formation of a negatively charged OP moiety on the active site serine that, in contrast to the non-aged OP-adduct, results in permanent inhibition wherein the enzyme cannot be reactivated [18]. While most OPs undergo aging via the loss of an O-alkyl side chain [19], certain classes of OP compounds, such as the phosphorodiamidates, undergo aging via a deprotonation step [20]. While the inhibition and aging of OP compounds on PNPLAs such as NTE (PNPLA6) have been studied extensively (reviewed in [15]), it is currently unclear whether OP compounds can undergo the aging reaction following their inhibition of the serine hydrolase activity of patatin.

Given the lack of an experimentally derived structure for the interaction of a PNPLA with inhibitors or a native lipid substrate, we explored whether interaction of patatin with OP compounds could serve as a model to better understand the structural basis for the interaction of PNPLAs with their native substrates as well as the transition states present during substrate hydrolysis. Thus, while the selenomethionine-derivatized structure of native pat17 has been published [6], there are currently no structures available for the native (non-derivatized) protein as well as the modes of inhibition and aging of an OP compound on patatin. To address these gaps in knowledge, we present a high-resolution crystal structure of pat17 in its native state (PDB ID 4PK9) as well as pat17 structures inhibited by methyl arachidonyl fluorophosphonate (MAFP; PDB ID 4PKB) and aged by DFP (PDB ID 4PKA), a known neuropathic OP compound.

## Results and Discussion

### Pat17 is inhibited by DFP and MAFP

Currently, little is known about the inhibitory potential of OP compounds such as DFP and MAFP toward patatin, beyond the fact that DFP inhibits the insecticidal ability of patatin [5]. Consistent with this observation, we found DFP is capable of inhibiting pat17 with a 20-min IC_50_ of 182.1±11.1 µM. Furthermore, the DFP adduct is capable of undergoing the aging reaction (a post-inhibitory reaction involving OP adduct gaining a net negative charge) with a pseudo-first-order rate constant of aging (*k_4_*) of 0.062±0.004 min^−1^ and a corresponding half-life of aging (*t*_1/2 aging_) of 11±0.38 min (Table 1). MAFP was found to be more potent against pat17 with a 20-min IC_50_ of 116±4.6 nM (Table 1). However, analysis of the potential of MAFP-inhibited pat17 to age revealed complete reactivation at all time points within the measured 20 minute interval, suggesting that unlike DFP, MAFP-inhibited pat17 does not age within the tested time frame (Table 1). However, the chiral nature of the MAFP and the use of a racemic mixture of the inhibitor did not allow for testing the potency or reactivation kinetics of the resolved stereoisomers of MAFP against pat17. These findings are discussed further in the section on the analysis of OP adducts on pat17.

**Table 1:** Kinetics of inhibition and aging of DFP and MAFP against Pat17.

Inhibitor	20 min IC_50_ (µM)^1^	*k*_4_ (min^−1^)	t_1/2 aging_ (min)
DFP	182.1±11.1	0.062±0.004	11.1±0.38
MAFP	0.116±0.0046	---2	---2

1 Mean 20-min IC_50_ ± SEM obtained from 2–4 independent experiments.

2 Complete reactivation occurred at all time points, thus no aging was observed within the 20-min interval.

### The overall structure of pat17 is a modified α/β hydrolase fold with a Ser77-Asp215 catalytic dyad

**Figure 1:**
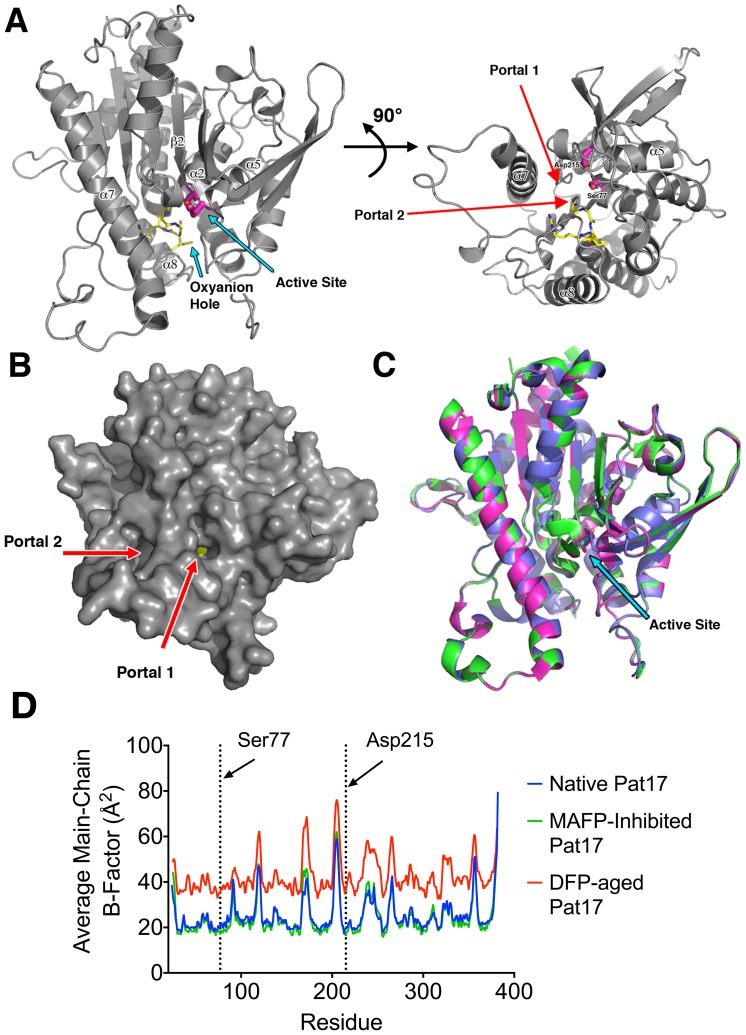
Pat17 structure. (A) The left panel is the overall view of pat17. The protein consists of 8 β-strands and 9 α-helices. The patatin fold is a modified α/β hydrolase fold with a central β-sheet sandwiched by α-helices. The residues that form the oxyanion hole (Gly37, Gly38, Ile39, Arg40 and Gly41) are colored yellow while residues in the active site dyad (Ser77 and Asp215) are shown in magenta. The right panel is a 90° rotation of the left panel about the x-axis depicting the location of the two portals (shown as red arrows) flanking helix 7 (α7) that lead into the active site chamber of the enzyme. (B) Solvent-accessible surface of native pat17 depicting the location of the two portals (denoted via red arrows). Pat17 is shown in the same orientation as the left panel in (A) with Ser77 rendered as yellow spheres. (C) Overlay of pat17 in its native (green), MAFP-inhibited (magenta) and DFP-aged (blue) states showing that there is no significant global conformational change associated with inhibition and aging of an OP compound on the active site serine (Ser77) of the enzyme (denoted as sticks with carbons colored green (native), magenta (MAFP-inhibited) or blue (DFP-aged)). An arrow denotes the active site of pat17. (D) Overlay of average main-chain B-factors for the residues in native, inhibited and aged pat17.

Pat17 purified and crystallized as a monomer in the asymmetric unit. Overall, our structure of native (non-derivatized) pat17 is similar to the SeMet-derivatized structure described previously by Rydel *et al*. [6] (RMSD  = 0.27 Å). In agreement with results presented by Rydel *et al*. [6], pat17 consists of 8 β-strands and 9 α-helices arranged in a modified α/β hydrolase fold with a central 6-stranded β-sheet consisting of five parallel and one anti-parallel β-strands sandwiched by 2 α-helices in the front and 7 α-helices in the back (Figure 1A left panel). The catalytic Ser77 is located on a loop between β2 and α2 (termed the nucleophilic elbow) that contains the classic G-X-S-X-G lipase consensus sequence motif [21]. Ser77 lies adjacent to Asp215 creating a catalytic dyad defined by Rydel *et al*. [6] (Figure 1A).

**Figure 2:**
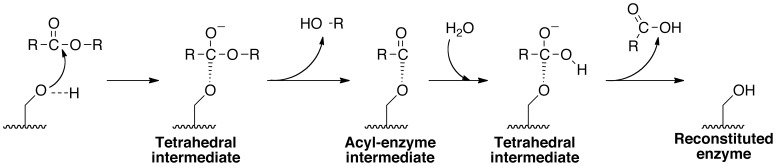
Proposed catalytic mechanism for the active site of Pat17. Asp215 (not shown) acts as a general base and activates the Ser77 nucleophile by abstracting its terminal hydrogen. The activated Ser77 (shown as –OH) attacks the acyl carbon of the substrate forming a tetrahedral intermediate whose negative charge is shielded by the oxyanion hole of Pat17 (not shown). Loss of R-OH yields an acyl-enzyme intermediate that is hydrolyzed rapidly (passing through another tetrahedral intermediate) to release the acyl moiety and regenerate the enzyme.

During catalysis, it is expected that Asp215 acts as a general base whose Oδ2 activates the Ser77 Oγ by abstracting its proton, turning Ser77 into a potent nucleophile in concert with its attack on the substrate (Figure 2) [6]. This is a similar mechanism to the one proposed for the active site dyad of cPLA_2_ (PDB ID 1cjy) [7] and the predicted Ser966-Asp1086 dyad in NTE [16]. In contrast, traditional serine hydrolases, such as the protease chymotrypsin and the esterase acetylcholinesterase (AChE), possess Ser-His-Asp/Glu triads [8], [9]. These catalytic triads work with the imidazole ring of the histidine (whose electronegativity is increased via hydrogen bonding with the aspartate/glutamate residue) acting as a general base to abstract the proton from the -OH group of the catalytic serine residue. This results in the activated serine being turned into a potent nucleophile that attacks the substrate resulting in the formation of an acyl-enzyme intermediate. The resulting acyl-enzyme intermediate is, in turn, hydrolyzed by a water molecule, leaving behind the reactivated enzyme and hydrolyzed substrate. In contrast, the catalytic dyad of pat17 functions with Asp215 acting as the general base that activates the catalytic Ser77 residue [6].

The catalytic dyad of pat17 sits at the bottom of a large chamber (153.6 Å^3^ calculated using VOIDOO [22]) lined with the following residues: Gly36, Gly37, Gly38, Ile39, Ser77, Thr78, Leu81, Phe105, Tyr106, Gly110, Phe114, Phe194, and Asp215. Two portals were found to flank helix 7 (α7) (Figure 1A right panel and B) with the first portal (portal1) being 16 Å in length and lined with the following residues: Ala190, Pro191, Ala 217, Val218, Gln288, Thr291 and Asp292. The second portal (portal2) is 15.2 Å in length and is lined with the following residues: Arg40, Gly261, Thr262, Lys289, Asp292, Ala293, Gln320, Leu324, Thr329 and Met331. The conserved binding pocket for the negatively charged phosphoryl oxygen that occurs as a result of aging (termed the oxyanion hole) is located on a loop between β1 and α1 encompassing residues Asp35, Gly36, Gly37, and Gly38 within 5 Å from Ser77’s Oγ (Figure 1A).

### Analysis of the OP adducts on Ser77

**Figure 3:**
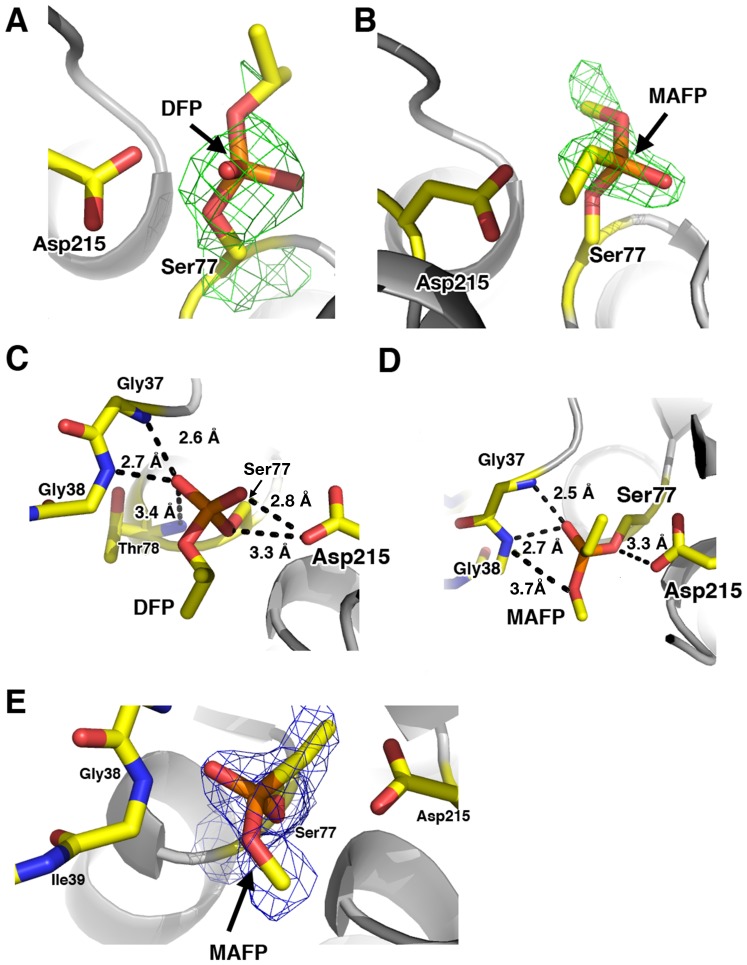
Pat17 bound to OPs. Initial difference (*F_o_-F_c_*) maps contoured at 3σ (depicted as a green mesh) showing the positive electron densities around (A) the aged DFP and (B) the non-aged MAFP adducts on the catalytic Ser77 residue of pat17. (C and D) The active site of patatin with (C) the aged DFP and (D) non-aged MAFP adducts showing contact distances between the adducts and the oxyanion hole of pat17 as well as the contact distance between the negatively charged oxygen atoms of the aged DFP and the backbone amide nitrogen of Thr78. For clarity, the orientation of the molecule is a 180° rotation about the X- and Y-axes in panel A. (E) 2F_o_-F_c_ electron density (contoured at 1 σ; depicted as a blue mesh) around the catalytic Ser77 residue in complex with MAFP. The active site Ser77 and Asp215 residues as well as the residues that comprise the oxyanion hole of pat17 are rendered as sticks with the following color scheme: yellow  =  carbon, blue  =  nitrogen and red  =  oxygen.

**Figure 4:**
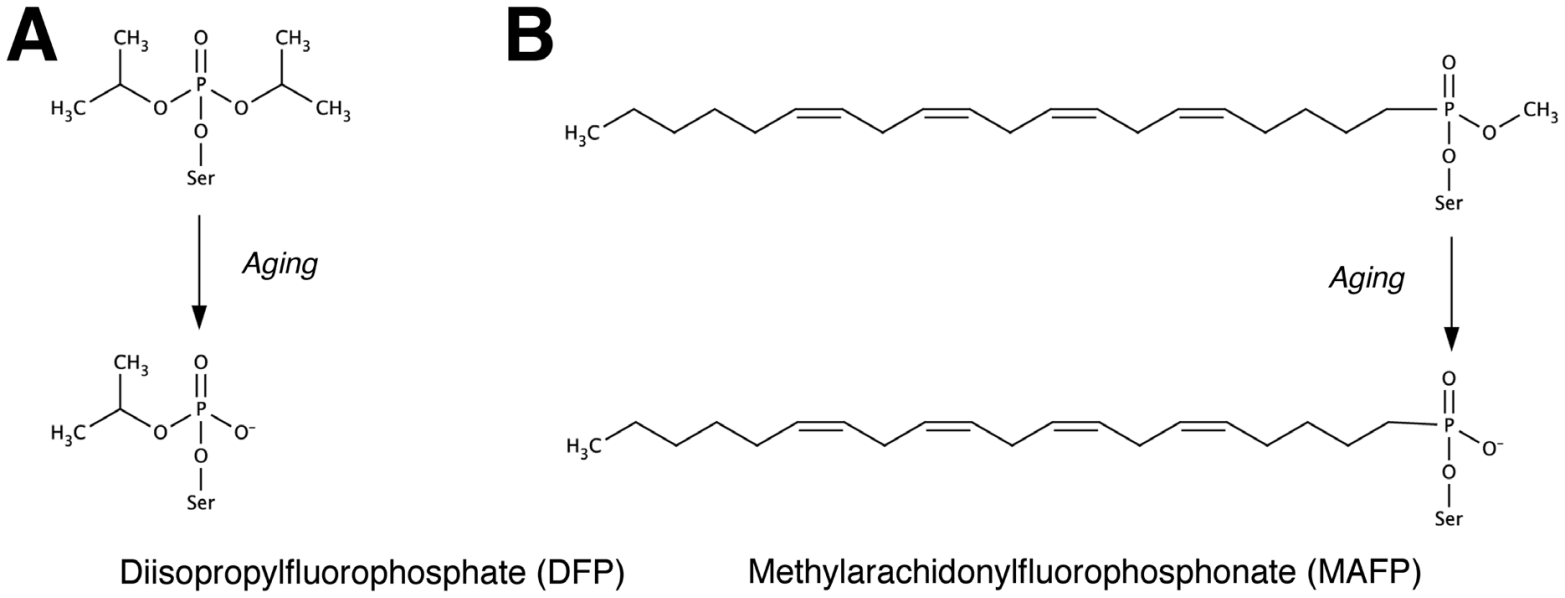
Structures of (A) DFP and (B) MAFP in their native state as well as the putative structures of these inhibitors in their aged forms. Chemical structures were generated in MarvinSketch 5.5 (ChemAxon, http://www.chemaxon.com).

Initial analysis of the difference (F_o_-F_c_) electron density maps of the DFP- and MAFP-adducted structures clearly showed the presence of phosphoryl adducts on the Ser77 nucleophile visible up to contour levels of 6σ (Figure 3A and 3B). The 2.6 Å structure of pat17 co-crystallized with DFP showed an aged adduct (Figure 3C) consisting of a serine-bound DFP moiety lacking an isopropyl chain (Figure 4A), indicating that aging had taken place. The DFP-aged structure contains a negatively charged dealkylated oxygen. This negatively charged oxygen was within hydrogen-bonding distance to the oxyanion hole (being located 2.6 Å, 2.7 Å and 3.4 Å from amide nitrogen atoms of Gly37, Gly38 and Thr78, respectively) and 5.1 Å from the Oδ2 of Asp215, which together with Ser77 comprises the catalytic dyad of patatin. Furthermore, the phosphoryl oxygen of DFP was located within hydrogen-bonding distance (2.8 Å) from the Oδ2 of Asp215 (Figure 3C).

Crystals of the DFP-pat17 complex were grown in a solution with a final pH of 5.4 (measured at 4°C). Computational analysis of the pKa of the dealkylated oxygen atom in the aged Ser77-DFP complex using MarvinBeans (ChemAxon) predicted it to be 1.9 (calculated for the Ser77-DFP complex in the absence of adjacent residues). Furthermore, predictions of the protonation state of the aged DFP-Ser77 conjugate in the context of the intact pat17 protein using YASARA and MOE revealed the dealkylated phosphoryl oxygen of the aged DFP adduct to be deprotonated at pH 5.4, a finding consistent with our pKa calculations and previously published pKa values for alkylphosphoric acids [23]. Consequently, based on our structure (Figure 3C), we propose that hydrogen bonding between this negatively charged oxygen and the protonated amide nitrogens of Gly37 and Gly38 help stabilize the negatively charged OP moiety within the active site chamber of pat17. This is expected to be similar to how the oxyanion hole helps stabilize the negatively charged tetrahedral intermediate of a native substrate during catalysis (Figure 2).

The 2.1 Å structure of the pat17–MAFP complex showed the presence of a non-aged (S)-MAFP moiety (Figure 3B and 3D) bound to pat17; i.e., the potentially labile O-methyl group was intact. This was in contrast to the aged DFP-moiety (Figure 4B), which lacked one of its O-isopropyl groups. While the inability of MAFP-inhibited pat17 to undergo aging may appear to be unusual (given that other phosphonate-inhibited serine hydrolases are capable of undergoing the aging reaction [24]), this finding is consistent with our analysis of the enzymology of pat17, wherein inhibition of the enzyme with MAFP was shown to be reactivatable within the tested 20 minute time-frame (Table 1). This is also consistent with previous findings from the crystal structures of group VIIA phospholipase A2 in complex with sarin, soman, and tabun (phosphonate agents that undergo aging following inhibition of AChE) that showed the phosphonyl OP adducts to be in their non-aged states [25].

MAFP is a chiral molecule with a stereocenter around the phosphorus atom. Interestingly, the observed MAFP adduct on the active site nucleophile (Ser77) of pat17 is of the S-enantiomer. Attempts to fit (R)-MAFP into the observed density around Ser77 resulted in steric clashes between the long acyl chain of MAFP and the wall of the active site chamber of pat17, further supporting the notion that the observed MAFP conjugate is of the (S)-enantiomer. This is similar to previous observations of MAFP in complex with fatty acid amide hydrolase (PDB ID 1MT5) [26], wherein the S-enantiomer was resolved in complex with the enzyme, despite the use of a racemic mixture of MAFP. The fact that (S)-MAFP is observed in complex with pat17 is suggestive of an increased potency or stability of the (S)-MAFP-pat17 complex over its (R)-counterpart. However, further studies are needed to establish the kinetics of stereospecific inhibition and aging of the resolved stereoisomers of MAFP against pat17 and its homologues.

Analysis of MAFP adduct on pat17 revealed that its phosphoryl oxygen is in close proximity (<3 Å) to the backbone amide nitrogens of Gly37 and Gly38, which form part of the oxyanion hole of pat17 (Figure 3D). Furthermore, when pat17 is in complex with (S)-MAFP, its Ser77 Oγ is located 3.3 Å from the Oδ2 of Asp215, while the O-methyl group of MAFP is positioned within the active site chamber. Additionally, the first alkyl carbon (C1) of the acyl chain of MAFP is located 2.8 Å from the Oδ2 of Asp215 (Figure 3D), in a similar orientation as that seen for the phosphoryl oxygen of the aged DFP adduct (Figure 3C). Interestingly, the alkoxy oxygen of (S)-MAFP is located 3.7 Å from the backbone amide nitrogen of Gly38 (Figure 3D). This is in contrast to the negatively charged dealkylated oxygen of the aged DFP adduct, which was located <3 Å from the backbone amides of the oxyanion hole. Taken together, these observations suggest that the protonated amides in the oxyanion hole of pat17 may play a role in the aging reaction by stabilizing the emerging net negative charge on the alkoxy oxygen during the dealkylation phase of the aging process. This is a similar role to that played by the oxyanion hole in the normal catalytic process and can be contrasted to the previously proposed role for the protonated form of His440 (the general base in the catalytic triad of AChE) in the aging of an OP adduct on the catalytic nucleophile (Ser200) of AChE [27]. As such, the putative preference of pat17 towards the S-enantiomer of MAFP could shield its labile O-methyl group from the residues that form the oxyanion hole of patatin, thus hindering the aging of the (S)-MAFP adduct.

**Figure 5:**
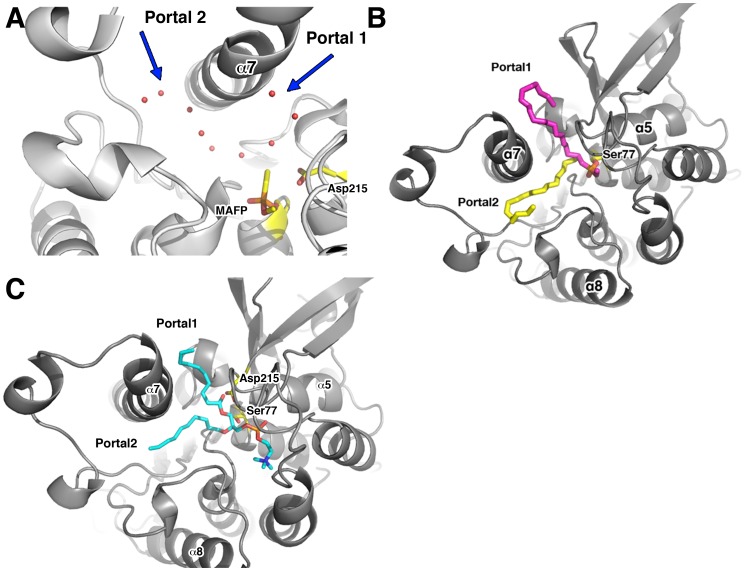
Substrate entry and exit points in Pat17. (A) Pat17 in complex with MAFP (with crystallographic water molecules shown as red spheres) depicting the trail of water molecules leading from the active site chamber through portals 1 and 2. (B) Model depicting the non-aged MAFP adduct with its arachidonic acid chain modeled as going through portal1 (with carbons colored magenta) or portal2 (with carbons colored yellow). (C) Model showing the phospholipid POPC (PDB ligand ID POV; depicted with carbons colored cyan) accommodated within the active site of pat17 with the lipid side-chains modeled within both, portal1 and portal2. The active site Ser77 and Asp215 residues as well as the MAFP and POPC moieties are rendered as sticks with the following color scheme: yellow/magenta/cyan  =  carbon, blue  =  nitrogen and red  =  oxygen.

The complete arachidonic acid chain of MAFP was not visible in the electron density maps, but trails of waters leading to portals 1 and 2 were apparent in the MAFP-bound pat17 structure. Based on this observation, we hypothesized that the acyl chain of MAFP could fit in either portal (Figure 5A). Fitting the acyl chain of MAFP to the observed water trails leading to portals1 and 2 failed to conclusively improve the observed electron density around the acyl chain of the MAFP moiety. Therefore, the phosphonate head of MAFP was fit to the visible density around Ser77 together with the first two alkyl carbons (C1 and C2) of the acyl chain of MAFP (Figure 3B). The remaining atoms of the acyl chain of MAFP were modeled to follow the two trails of water molecules with the results shown in Figure 5B.

Analysis of the electrostatic surface potentials of the two portals (as analyzed via the APBS module in PyMOL [28]) revealed portal2 to be more hydrophobic than portal1. Therefore, we propose that there could be a preference for the long acyl chain of MAFP to be contained within portal2. However, given that the electron density was not visible for certain regions of the acyl side chain of MAFP (Figure 3E), as well as the fact that MAFP used in this study comprised unresolved enantiomers, it is unclear which portal (1 or 2) contains the long acyl side-chain of MAFP.

Moreover, the increased hydrophobicity of MAFP (due to the presence of a 20-carbon atom acyl chain), could explain its increased potency over DFP for pat17, because patatin, as a lipase [2], would be expected to have a preference for lipids or lipid-like substrates containing hydrophobic fatty acid chains. Consistent with this, we found that MAFP was 1575-fold more potent than DFP as an inhibitor of pat17. Taken together, these considerations suggest that OP compounds with increasingly hydrophobic side chains would have an increased inhibitory potency against patatin.

Physiologically, it is possible that during its normal catalytic cycle, pat17 is able to accommodate a fatty acid chain in either of its portals, allowing for the facile release of a fatty acid chain during catalysis. This possibility is supported in part by the fact that the phospholipid 1-palmitoyl-2-oleoyl-sn-glycero-3-phosphocholine (POPC; PDB Ligand ID POV) can be modeled into the active site chamber of pat17, with each lipid chain accommodated within both portals (Figure 5C).

### No substantial global conformational changes are associated with either inhibition or aging of an OP compound on Pat17

As seen in Figure 1C, comparison of the DFP-aged structure of pat17 and the MAFP-inhibited structure to the native pat17 structure yielded Cα backbone RMSD values of 0.19 Å (DFP- aged vs. native) and 0.12 Å (MAFP-inhibited vs. native). These findings suggest that there are no substantial overall conformational changes (i.e. all RMSD changes were below 1 Å, the generally accepted range for the deviation between two identical structures) in the Cα backbone associated with either inhibition or aging of OP compounds on pat17. Furthermore, analysis of the average main-chain temperature factors failed to highlight any pronounced peaks or valleys in the B-factor plots that were unique to either the MAFP-inhibited or DFP-aged pat17 relative to the native pat17 structure (Figure 1D). However, the overall temperature factors for pat17 in complex with DFP were higher than those seen in its native or MAFP-conjugated counterparts (Figure 1D and Table 2), presumably due to the lower resolution of this structure compared to the structures of native and MAFP-bound pat17.

**Table 2:** Crystallography Data Collection and Refinement Statistics.

Data Set	Patatin 17 + DFP (inhibited and aged)	Patatin 17 + MAFP (inhibited)	Pat17 (native)
PDB ID	4PKA	4PKB	4PK9
Space group	P4_3_2_1_2	P4_3_2_1_2	P4_3_2_1_2
Unit Cell (Å, °)	a = b = 52.99; c = 242.77; α = β = γ = 90	a = b = 53.24; c = 245.01; α = β = γ = 90	a = b = 53.12; c = 244.95; α = β = γ = 90
Wavelength (Å)	0.97626	0.97856	0.97856
Resolution (Å)	50.0–2.6	50.0–2.09	50.0–1.96
R_sym_ (%)^1^^,^ 2	5.3 (41.7)	5.6 (10.4)	7.6 (27.4)
<I/sI>^3^	20 (3)	20(20)	20 (5)
Completeness (%)^4^	95.3 (87.8)	99.0 (99.7)	93.2 (76.4)
Redundancy	6.4 (5.6)	4.6 (5.0)	5.1 (3.2)
Solvent Content	41.6	41.9	41.8
Molecules per asymmetric unit	1	1	1
**Refinement Statistics**			
Resolution (Å)	2.6	2.09	1.96
σ cutoff [*F*/σ(*F*)]	0	0	0
R-Factor (%)^5^	21.3	17.8	19.4
R_free_ (%)^6^	27.8	21.2	22.4
Water Molecules	37	158	127
Unique Reflections	10963	21776	24814
R.m.s.d.^7^			
Bonds (Å)	0.009	0.01	0.01
Angles (°)	1.06	0.97	0.99
Mean B-value (Å^2^)	45.4	28.2	28.9
Molprobity Score (All Atom Contacts)^8^	3.09	3.63	1.6

1 Statistics for highest resolution bin of reflections in parentheses.

2 R_sym_  = S_h_S_j_ l I_hj_-<I_h_> l/S_h_S_j_I_hj_, where I_hj_ is the intensity of observation j of reflection h and <I_h_> is the mean intensity for multiply recorded reflections.

3 Intensity signal-to-noise ratio.

4 Completeness of the unique diffraction data.

5 R-factor  =  S_h_ I IF_o_I – IF_c_I I/S_h_IF_o_I, where F_o_ and F_c_ are the observed and calculated structure factor amplitudes for reflection h.

6 R_free_ is calculated against a 10% random sampling of the reflections that were removed before structure refinement.

7 Root mean square deviation of bond lengths and bond angles.

8 Number of steric overlaps>0.4 Å per 1000 atoms.

In keeping with the lack of an overall conformational change, comparison of the Cα backbone RMSDs between the residues that form the active site chamber of pat17 in the native, MAFP-inhibited and DFP-aged states revealed no substantial localized conformational changes with RMS deviations below the 1 Å threshold (RMSD  = 0.13 Å (DFP-inhibited and aged vs. native) and 0.25 Å (MAFP inhibited vs. native)). The lack of a substantial conformational change (either global or localized within the active site region) between the native and MAFP-inhibited pat17 structures is consistent with findings from the recently described structures of group VIIa phospholipase-A2 in complex with the non-aged forms of DFP and sarin (PDB IDs 3f9c, 3f96 respectively) [25].

With respect to aging, previous findings from the X-ray crystal structures of murine AChE in complex with the aged and non-aged forms of DFP, methamidophos, and fenamiphos revealed a conformational change in the acyl loop region of the enzyme [29]. In contrast to these findings with AChE, our structures of the inhibited and aged forms of pat17 revealed no such localized changes, with the exception of Met331, in either the aged or inhibited states (Figure 1). The side-chain rotomer of Met331 is conformationally flexible and the presence of the alkyl side chains of DFP and MAFP stabilize distinct conformations of this residue. This suggests that unlike AChE, inhibition and aging of OP compounds on patatin and other PNPLAs may proceed without significant perturbations in their overall structures.

### Summary

In this study, we demonstrated that pat17 could be inhibited and aged by OP compounds like DFP. We also show that certain OP compounds, like MAFP do not undergo aging, while still possessing the ability to inhibit pat17, a finding confirmed via structural analysis using x-ray crystallography.

The structure of pat17 in complex with the aged DFP moiety showed the phosphoryl oxygens of DFP to be within hydrogen-bonding distance to its oxyanion and Asp215 of the catalytic dyad (Figure 3). This finding provides a structural model for how the tetrahedral intermediate moieties of a phospholipid substrate could be stabilized within the active site chamber of patatin and suggests a role for the oxyanion hole of pat17 in stabilizing the emerging negative charge on the alkoxy oxygen of the dealkylating OP adduct during the aging reaction. We also describe the presence of two portals leading to the active site chamber of pat17 that could accommodate the lipid acyl chain of MAFP. Given that the arachidonic acid chain of MAFP could be modeled into both portals suggests that the acyl chains of phospholipids could be accommodated within either portal, with the more hydrophobic one (portal2) presumed to be the favored location.

The results of this investigation also provide insights into the putative structural basis for the interaction of organophosphorus compounds with NTE (PNPLA6), which have been characterized extensively via biochemical techniques (reviewed in [30]). It is currently thought that the aging of an OP compound on the active site nucleophile of NTE (Ser966) is a critical component in triggering a delayed axonopathy termed OP-compound induced delayed neurodegeneration (OPIDN). The fact that aging of an OP compound on pat17 fails to induce a global conformational change in the protein suggests that a similar effect may be observed within the catalytic domain of NTE, which has previously been shown to share structural homology with pat17 [16]. Therefore, the functional effects of aging of an OP on NTE, its role in initiating OPIDN as well as altering interactions between NTE and its natural substrates or binding partners remain areas for further investigation.

In conclusion, this investigation demonstrates for the first time that OP compounds can both, inhibit pat17 while also undergoing the post-inhibitory aging reaction. Furthermore, we describe the first structures for OP compounds in complex with a PNPLA, thereby providing templates for further structural and functional investigations into the interaction of these enzymes with their inhibitors and substrates.

## Materials and Methods

### Supplies

Unless otherwise indicated, all reagents were purchased from Sigma-Aldrich (St. Louis, MO). DFP was purchased from Calbiochem (San Diego, CA). MAFP was purchased from Cayman Chemical (Ann Arbor, MI). All buffers were pH-adjusted at room temperature (22°C).

### Protein purification

The pat17 expression plasmid was kindly supplied by the Monsanto Company (Chesterfield, MO) and contained pat17 (residues 24-386) with a non-cleavable N-terminal hexa-histidine tag (MHHHHHHAMA) [6]. The expressed his-tagged pat17 was purified via a modification of the procedure described by Rydel *et al*. [6]. The pat17 plasmid was transformed into *Escherichia coli* CD41 cells. The cells were grown at 37°C in 2xYT media containing 30 µg/L kanamycin to an OD_600_ of 0.6, induced with 0.4 mM isopropyl β-D-1-thiogalactopyranoside and the protein was expressed overnight at 25°C. The cells were harvested via centrifugation at 6000×g for 10 min at 4°C and stored at -80°C until needed.

For purification of pat17, *Escherichia coli* CD41 cells were lysed via sonication in a buffer containing 50 mM Tris-HCl pH 8.5 and 150 mM NaCl. Cellular debris was removed via centrifugation at 20000×g for 45 min at 4°C. The supernatant was applied to a Ni-NTA column (Invitrogen, Carlsbad, CA) and pat17 was eluted with 50 mM Tris-HCl pH 8.5, 150 mM NaCl, and 300 mM imidazole at 4°C. The eluted protein was dialyzed overnight against 25 mM Tris-HCl pH 7.5 at 4°C and purified to homogeneity via size exclusion chromatography on a Sephedex-75 column equilibrated with 25 mM Tris pH 7.5. The pat17-containing fractions were pooled and dialyzed overnight against 10 mM Tris-HCl pH 7.4 at 4°C. Prior to crystallization, pat17 was concentrated to 10 mg/mL using an Amicon ultra-15 concentrator with a 10000 Da molecular-mass cut-off (Millipore, Billerica, MA).

### Protein crystallography

Tetragonal crystals of native pat17 were grown via vapor diffusion at 20°C using the hanging-drop method. Drops comprised equal volumes of the protein concentrated to 10 mg/mL in 10 mM Tris-HCl pH 7.4 and precipitant solution (0.1 M sodium acetate pH 4.6, 49% (v/v) polyethylene glycol-400 and 225 mM cesium chloride). Crystals of native pat17 grew within two weeks to a maximum size of 0.1 mm×0.1 mm×0.05 mm in space group P4_3_2_1_2 with a unit cell a  =  b  = 53.32 Å, c  = 244.95 Å; α  =  β  =  γ  = 90° (Table 2). Crystals were cryoprotected using the well solution prior to data collection.

Crystals of pat17 inhibited with MAFP were obtained by soaking crystals of native pat17 in a precipitant solution containing 0.1 M sodium acetate pH 4.6, 49% (v/v) polyethylene glycol-400, 225 mM cesium chloride and 1.3 µM MAFP for 24 h. Crystals were harvested directly from the soak solution and flash-frozen in liquid nitrogen prior to data collection. Diffraction data for the native and MAFP- inhibited pat17 crystals were collected at the Advanced Photon Source (beamline 21-ID-G; Argonne, IL) using a Mar-300 CCD at −180°C at a wavelength of 0.97856 Å (Table 2). The resulting data were processed using the HKL2000 suite [31]. Analysis of the diffraction data revealed the crystals to be in space group P4_3_2_1_2 (a  =  b  = 53.24 Å, c  = 245.01 Å; α  =  β  =  γ  = 90°). The Matthews coefficient and approximate solvent content of the crystal were calculated in CCP4.

Crystals of the Pat17-DFP complex occurred via co-crystallization experiments. Pat17 was mixed with 1 mM DFP for 48 hours at 4°C, then concentrated to 10 mg/mL in 10 mM Tris-HCl pH 7.4 prior to crystallization. Drops containing equal volumes of protein and precipitant (0.1 M sodium acetate pH 4.6, 46% (v/v) polyethylene glycol-400 and 75 mM ammonium sulfate) were setup at 4°C using the hanging-drop vapor diffusion method. Crystals grew within two weeks to a maximum size of 0.1 mm×0.1 mm×0.1 mm prior to being harvested and flash-frozen in liquid nitrogen. Diffraction data were collected at the Advanced Photon Source (beamline 21-ID-D; Argonne, IL) using a Mar-300 CCD at −180°C. 360 degrees of data were collected at a wavelength of 0.97626 Å to 2.6 Å resolution (Table 2). The resulting data were processed using the HKL2000 suite. Analysis of the diffraction data revealed the crystals to be in space group P4_3_2_1_2 (a  =  b  = 52.99 Å, c  = 242.77 Å; α  =  β  =  γ  = 90°). The Matthews coefficient and approximate solvent content of the crystal were calculated in CCP4.

The structures of native, DFP-aged and MAFP-inhibited pat17 were phased via molecular replacement with Phaser [32] using the structure of selenomethionine-derivatized patatin (PDB ID 1OXW) as the search model. The resulting structures for DFP-aged and MAFP-inhibited pat17 were refined in the Crystallography and NMR System (CNS) [33] via an initial round of rigid body refinement followed by subsequent rounds of simulated annealing and B-factor refinement. Once the protein was refined, the Ser77 adducts were placed in Fo–Fc electron density using O [34] and subsequently refined with CNS. The coordinates and CNS parameter and topology files for the monoisopropylphosphoryl-serine adduct were downloaded from the Hetero-compound Information Centre (HIC-UP) [35]. The coordinates for the MAFP-bound serine (MAY) were obtained from the structure of fatty acid amide hydrolase adducted to non-aged MAFP (PDB ID 1MT5 [26]) and the CNS parameter and topology files generated using the Dundee PRODRG2 server [36]. The native pat17 structure was refined via repeated rounds of restrained maximum likelihood refinement with TLS in Refmac [37]. Following each round of refinement, the structure was fitted using Coot [38]. A final round of geometry optimization in Refmac was undertaken for the DFP-bound structure while a final round of refinement using Buster-TNT [39] was undertaken for the native and MAFP-bound pat17 structures.

Each of the refined structures was analyzed using PROCHECK [40] and MolProbity [41]. Analysis of the Ramanchandran plot for each structure confirmed all residues to be in the allowed regions. The final pat17 structures were visualized in PyMOL (Schrödinger, New York, NY).

pKa calculations to predict the protonation state of the active site nucleophile of pat17 (Ser77) in complex with the aged DFP adduct were undertaken using Marvin Beans (ChemAxon). For all calculations, the backbone atoms of the DFP-bound serine moiety were capped with ACE and NME in YASARA Structure (ver. 14.6.23; YASARA Biosciences) to mimic the presence of a longer protein chain. The results of this pKa calculation were further verified via *in silico* prediction of the protonation state of the dealkylated phosphoryl oxygen of the aged DFP moiety in the context of the entire pat17 protein using YASARA Structure and MOE with the environmental pH set at 5.4.

### Pat17 inhibition

The inhibitory potencies of DFP and MAFP against pat17 were assessed spectrophotometrically as the change in rate of phenyl valerate hydrolase activity. The spectrophotometric assays were performed as described previously for NTE [42]. All experiments were repeated 3–4 times. Pat17 was diluted in 10 mM Tris-HCl pH 7.4 to a point where the uninhibited pat17 activity yielded a reading of less than 1 absorbance unit at 486 nm (at 37°C). 40 µL of the diluted pat17 was titrated with varying concentrations of inhibitor (DFP or MAFP; 10 µL inhibitor dissolved in DMSO with final [DMSO] <1% by volume). Following an incubation period of 20 min at 37°C, 100 µL of the substrate (5.26 mM phenyl valerate/dimethylformamide/0.03% (v/v) Triton X-100) was added to the enzyme and allowed to incubate for 20 min at 37°C. The reaction was stopped by the addition of 100 µL of 1.23 mM 4-aminoantipyrine (4-AAP) in 9.5 mg/mL sodium dodecyl sulfate (SDS) after which the color was developed via the addition of 50 µL of 2.1 mM potassium ferricyanide. The chromophore was allowed to develop for 5 min and read at 486 nm on a SpectraMax plate reader (Molecular devices, Sunnyvale, California). The 20-min IC_50_ values of MAFP and DFP against pat17 were determined via linear regression; -log_10_([inhibitor]) was plotted against % inhibition. The resulting linear regression equation was used to calculate the [DFP] and [MAFP] at which 50% inhibition was obtained. All data was analyzed in GraphPad Prism (version 6.0e).

### Aging of OP inhibited pat17

Aging of patatin in the presence of DFP and MAFP was assessed in 50 mM Tris-HCl pH 7.4 as described earlier for the catalytic domain of NTE [20]. Pat17 was inhibited with either 4 mM DFP or 4 µM MAFP for 3 min at 37°C after which each aliquot was diluted 1∶2000 (v/v) with 50 mM Tris-HCl pH 7.4 to stop the inhibition reaction. The inhibited enzyme was allowed to age for timed intervals ranging from 0 to 20 min. Aliquots of inhibited enzyme solution were incubated with 2-PAM (final concentration 100 µM) for 20 min at 37°C at the end of each time point. Following incubation with 2-PAM, the residual enzyme activity was measured. The residual activity of the enzyme in the absence of 2-PAM was assessed as the control [20].

The apparent first-order rate constants of aging (*k*_4_) for DFP and MAFP against pat17 were assessed via linear regression; ln(100/% reactivation) was plotted versus time. The slope (β_1_) of the resulting linear regression equation was equal to the *k*_4_ with the half-life of aging (t_1/2_) being equal to ln(2)/*k*_4_ [18], [43]. All experiments were repeated 2–3 times and the data were analyzed in GraphPad Prism (version 6.0e).

### Statistical Analysis

All statistical analyses were undertaken using GraphPad Prism (ver 6.0e). The steady-state 20-minute half maximal inhibitory concentrations (IC_50_) for DFP and MAFP against pat17 as well as the first-order rate constants of aging for DFP are presented as mean ± SEM.

Root mean square deviation (RMSD) values between the described crystal structures relative to native pat17 were calculated in PyMOL. Average main chain temperature factors for each residue in the native, DFP-aged and MAFP-inhibited pat17 structures were calculated in CCP4 with subsequent visualization and analysis undertaken in GraphPad Prism.
